# SMN complex member Gemin3 self-interacts and has a functional relationship with ALS-linked proteins TDP-43, FUS and Sod1

**DOI:** 10.1038/s41598-019-53508-4

**Published:** 2019-12-10

**Authors:** Rebecca Cacciottolo, Joanna Ciantar, Maia Lanfranco, Rebecca M. Borg, Neville Vassallo, Rémy Bordonné, Ruben J. Cauchi

**Affiliations:** 1 Institut de Génétique Moléculaire de Montpellier, CNRS-UMR 5535, Université de Montpellier, Montpellier, France; 2 Department of Physiology and Biochemistry, Faculty of Medicine and Surgery, University of Malta, Msida, Malta; 3 Centre for Molecular Medicine and Biobanking, Biomedical Sciences Building, University of Malta, Msida, Malta

**Keywords:** Epistasis, Amyotrophic lateral sclerosis

## Abstract

The predominant motor neuron disease in infants and adults is spinal muscular atrophy (SMA) and amyotrophic lateral sclerosis (ALS), respectively. SMA is caused by insufficient levels of the Survival Motor Neuron (SMN) protein, which operates as part of the multiprotein SMN complex that includes the DEAD-box RNA helicase Gemin3/DDX20/DP103. *C9orf72*, *SOD1*, *TDP-43* and *FUS* are ranked as the four major genes causing familial ALS. Accumulating evidence has revealed a surprising molecular overlap between SMA and ALS. Here, we ask the question of whether *Drosophila* can also be exploited to study shared pathogenic pathways. Focusing on motor behaviour, muscle mass and survival, we show that disruption of either TBPH/TDP-43 or Caz/FUS enhance defects associated with Gemin3 loss-of-function. Gemin3-associated neuromuscular junction overgrowth was however suppressed. Sod1 depletion had a modifying effect in late adulthood. We also show that Gemin3 self-interacts and *Gem3*^*ΔN*^, a helicase domain deletion mutant, retains the ability to interact with its wild-type counterpart. Importantly, mutant:wild-type dimers are favoured more than wild-type:wild-type dimers. In addition to reinforcing the link between SMA and ALS, further exploration of mechanistic overlaps is now possible in a genetically tractable model organism. Notably, Gemin3 can be elevated to a candidate for modifying motor neuron degeneration.

## Introduction

Motor neuron disease (MND) encompasses a seemingly heterogeneous group of neurological conditions that are nonetheless characterised by muscle weakness and paralysis thought to arise from the selective degeneration of motor neurons. Genetic factors play a major role in disease pathogenesis and the knowledge that mutations in genes encoding RNA-binding proteins (RBPs) can lead to MND, underscores RNA dysregulation as a key contributor to motor dysfunction^1–4^. In infants, the predominant MND is spinal muscular atrophy (SMA), typically an autosomal recessive condition caused by inactivating mutations in the *survival motor neuron 1* (*SMN1*) gene that are partly counteracted by the paralogous *SMN2* gene. Rather than total loss of the *SMN1-* or *SMN2*-encoded SMN protein, SMA is the result of insufficient SMN levels^5^. SMN, operating as part of a large multiprotein complex that includes Gemins 2–8 and Unrip, is indispensable for chaperoning the assembly of spliceosomal small nuclear ribonucleoproteins (snRNPs)^6,7^, in addition to a possible role in the assembly and axonal trafficking of messenger ribonucleoproteins (mRNPs) in motor neurons^8^. In adults, the most common MND is amyotrophic lateral sclerosis (ALS), which can be inherited (~10%) but is mostly sporadic (~90%). *Chromosome 9 open reading frame 72* (*C9orf72*), *Cu/Zn superoxide dismutase 1* (*SOD1)*, *transactive response DNA binding protein* (*TARDBP*) and *fused in sarcoma* (*FUS*), in that order, are ranked as the four most common genes causing familial ALS and mutations in these genes are increasingly detected in sporadic cases^9,10^. TAR DNA binding-protein 43 or TDP-43 (encoded by *TARDBP*), and FUS are RBPs that are involved in multiple levels of RNA processing^11,12^.

Although SMA and ALS are traditionally considered as separate MNDs, a notion supported by differences in genetic aetiology, disease onset and type of affected motor neurons, accumulating evidence has revealed a surprising overlap at a molecular level. First, SMN and/or SMN complex members are components of the interactomes of SOD1^13^, TDP-43^14,15^, FUS^16–19^ or the dipeptide repeat (DPR) proteins resulting from hexanucleotide repeat expansion in the *C9orf72* gene^20^. In addition, both TDP-43 and FUS were reported to localise to gems^14,15^, which are nuclear bodies enriched in SMN complexes^7,21,22^. Second, both diseases are characterised by disrupted RNA processing including snRNP perturbation^6,15,18,23–27^ and axonal transport defects^8,17,28–30^. Third, both ALS and SMA were found to co-occur within families^31^. Fourth, an abnormal change in *SMN1* copy number including gene deletion or duplication increases susceptibility to sporadic ALS^32–34^, presumably because deviations from normal SMN protein levels render motor neurons more vulnerable to degeneration. In corroboration, SMN deficiency was found to accelerate phenotypic severity in mutant *SOD1* mice^13^. Fifth, and most important, a depleted number of gems resulting from SMN reduction was identified as a signature feature of ALS in addition to SMA^35^. A follow-up study unexpectedly showed that motor neurons derived from SMA or ALS patients have heterogeneous SMN levels with those having low levels being highly susceptible to cell death^36^. This observation explains why increasing SMN levels was found to be beneficial not only to SMA^37,38^ but also to ALS, at least, in SOD1^39,40^ and TDP-43^41^ mouse models. Whether SMA therapeutics elevating SMN levels are also effective in ALS patients still remains to be determined.

Known and unknown components of molecular pathways can be uncovered in an unbiased fashion via genetic approaches. *Drosophila* has emerged as a premier model system for this task in view of its genetic tractability^42,43^. Indeed, genome-wide screens in *Drosophila* have yielded several modifier genes that are relevant to the pathology underlying either SMA^44–47^ or ALS^48–52^. However, the overlap has been surprisingly minimal and one study even reported that overexpression or RNAi-mediated knockdown of SMN failed to modify human FUS (hFUS)-induced neurodegeneration in *Drosophila* eyes^23^. This is in contrast to earlier findings in a cell-based system showing that overexpression of SMN rescued axonal defects induced by mutant FUS^17^. Although a common pathway uniting SMA and ALS could have developed later in evolution, it is highly likely that the screenable phenotype used in *Drosophila*-based investigations was not adequate to uncover interactions between SMA- and ALS-linked proteins. Therefore, the question of whether *Drosophila* can be exploited to study the shared pathogenic pathway linking SMA and ALS remains. Here, we address this question by using a different approach. First, instead of SMN, we focus on Gemin3, which is a core member of the SMN complex^7^. Our rationale is based on accumulating evidence that has essentially shifted the limelight from SMN to its Gemin associates revealing, a previously undisclosed, starring role in the operations of the SMN complex^6,53^. Second, we probe for a modifying effect in muscle, a tissue that is increasingly considered as a primary site of pathogenesis in both SMA and ALS^54–59^.

Gemin3, also known as DDX20 or DP103, is a DEAD-box RNA helicase which is involved in multiple cellular processes^60^. Most documented are its roles in RNA metabolism, including snRNP biogenesis where it functions within the SMN complex. To this end, we have recently shown that, in *Drosophila*, Gemin3 interacts both genetically and physically with pICln and Tgs1, two fundamental players in the snRNP biogenesis cycle^61^. Here, we extend the functional relationship to three key proteins linked to ALS. Hence, we demonstrate that a combination of *Gemin3* and *TDP-43* or *FUS* disruption exacerbates viability defects, motor dysfunction and muscle atrophy whilst suppressing neuromuscular junction (NMJ) overgrowth. Loss of Sod1 function is also responsible for inducing a prominent motoric decline in *Gemin3* mutant flies at a late stage in adult life. The likely explanation is an interference in a common pathway. Additionally, we show that Gemin3 is capable of self-binding and *Gem3*^*ΔN*^, a helicase domain deletion mutant, enhances the association when bound to wild-type Gemin3, an observation that offers an explanation for its dominant-negative mechanism of action. Collectively, our data reinforce the link between SMA and ALS in addition to giving impetus to further studies on the shared mechanisms in a genetically tractable model organism.

## Results

### Overexpression of human *TDP-43* in a *Gem3* mutant background induces adult lethality

**Figure 1:**
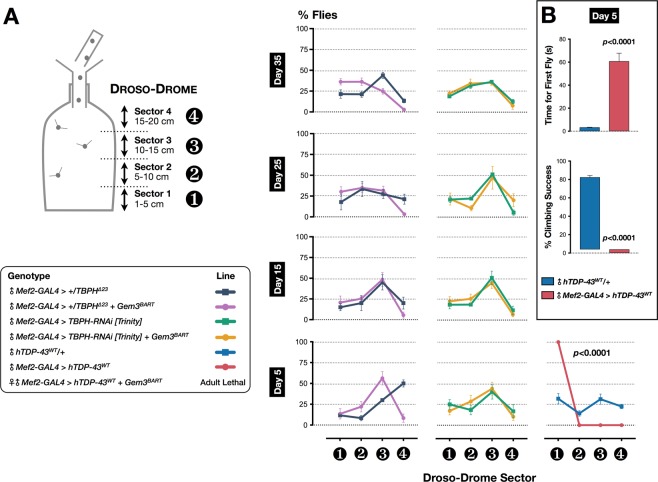
Gain-of-function identifies TDP-43 as a modifier of survival in Gem3^BART^-expressing flies. (**A**) *Left:* Flight performance was assessed by the Droso-Drome apparatus, where determination of flight capacity is based on which sector flies land after they are introduced at the top. *Right*: Removal of one copy (+/*TBPH*^*Δ23*^) or RNAi-mediated knockdown (*TBPH-RNAi [Trinity]*) of *TBPH* does not impair the motoric ability of flies with pan-muscular expression of Gem3^BART^. Indeed, at all time points, no significant differences were observed between the two groups in the number of flies that had no flight ability (sector 1) compared to those that retained the ability to fly (sectors 2–4). In contrast, ectopic expression of wild-type human TDP-43 (hTDP-43^WT^) in muscle impairs flight as early as day 5 post-eclosion. Flies were non-fliers, hence they all fell in sector 1. (**B**) Climbing success rate of flies with muscle-specific hTDP-43 overexpression was drastically reduced compared to control animals. Furthermore, assessment of the time taken for the first fly to reach a pre-set threshold determined that flies took significantly longer to attain this goal in contrast to the control genotype. In (**A,B**), data presented are the mean ± S.E.M. of at least 4 independent experiments, and for each time point measured, *n* ≥ 60 per genotype. Symbols indicate the sex of the genotype assessed: ♂ = males, ♀ = females, and ♀♂ = males + females. Significance as tested by two-way ANOVA, followed by Bonferroni’s *post hoc* tests (**A**) and the unpaired *t*-test (**B**) is indicated by the exact *p*-value.

Similar to SMN^45,62,63^, loss of Gemin3 impacts adult viability and induces motor dysfunction^64–66^. In addition to Gemin3, a select number of SMN complex components, including Gemin2, Gemin4, Gemin5, Gemin8 and Unrip, are required for neuromuscular function and survival in *Drosophila*^61,64,67^. It is therefore highly plausible that SMA is triggered by any perturbation in the stoichiometry of the SMN complex. We have recently isolated *Gem3*^*BART*^, a hypomorphic version of the *Gem3*^*ΔN*^ mutant, which lacks the N-terminal helicase core. Subsequently, we reported that alterations in the levels of SMN complex components precipitate the viability and motor phenotypes of *Gem3*^*BART*^ adult flies^68^. A similar outcome was observed on disruption of snRNP biogenesis factors pICln and Tgs1^61^. We wished to investigate whether a functional interaction also extends to Gemin3 and ALS-linked TDP-43. Missense mutations in this protein have been identified in 5% of familial and <1% of sporadic ALS cases^10^. TDP-43, an evolutionarily conserved protein, comprises of 2 RNA recognition motifs (RRMs), a nuclear localisation signal and a nuclear export sequence that mediate nuclear shuttling, as well as a C-terminal glycine-rich region where the majority of ALS-associated mutations occur^11^. Importantly, truncated TDP-43 is mislocalised from its predominantly nuclear location to ubiquitin-containing cytoplasmic inclusions in neurons of both sporadic and most familial forms of ALS^10,69,70^. Loss of TDP-43 nuclear function has been proposed as a primary mechanism linking TDP-43 proteinopathy to neuromuscular degeneration in ALS. In this context, we first asked whether decreased levels of TDP-43 can modify Gem3^BART^ phenotypes. We note that neither haploinsufficiency (*TBPH*^*Δ23*^) nor RNAi-induced knockdown (*TBPH-RNAi*) of *TBPH*, the *Drosophila TDP-43* homologue, had any effect on motor and viability phenotypes in flies with muscle-restricted *Gem3*^*BART*^ expression (Fig. 1, Table 1 and data not shown).

**Table 1:** Alleles of ALS-linked genes investigated in this study and their effect on viability when expressed either alone or in combination with *Gem3*^*BART*^ in muscle tissue.

ALS GENE	ALLELE	REF.	VIABILITY	
			*Mef*2*-GAL4>*	*Mef2-GAL4* > + *Gem3*^*BART*^
**Hs:** ***TDP-43*;** **Dm:** ***TBPH***	LOF: +/*TBPH*^*Δ23*^	^73^	Adult Viable	Adult Viable
	LOF^1^: *TBPH-RNAi [Trinity]*	73	Adult Viable	Adult Viable
	LOF^1^: *TBPH-RNAi [Merton]*	73	Adult Viable	Adult Viable
	LOF^1^: *TBPH-RNAi [Maudlin]*	126	Death at P	Death at P
	OE^2^: *hTDP-43*^*WT*^	72	Adult Viable	Death at P
	OE^1^: *hTDP-43*^*WT*^*.GFP*^*#10*^	72	Death at P	Death at L3
	OE^1^: *hTDP-43*^*WT*^*.GFP*^*#16*^	72	Death at P	Death at L3
	OE^1^: *hTDP-43*^*CTF*^*.GFP*^*#14*^	72	Death prior to L3	N/A
	OE^1^: *Flag.hTDP-43*^*WT*^	73	Death at P	Death at L3
	OE^1^: *Flag.TBPH*^*WT*^	73	Death prior to L3	N/A
	OE^2^: *TBPH*^*WT*^	75	Death prior to L3	N/A
	OE^2^: *Venus-TBPH*^*WT*^	75	Death at P	Death prior to L3
**Hs:** ***FUS*; Dm:** ***Caz***	LOF: *caz*^1^/+*	75	Adult Viable	Adult Viable
	LOF^2^: *caz-RNAi [Kellogg]*	121,127	Adult Viable	Adult Viable
	LOF^2^: *caz-RNAi [Oriel]*	N/A	Adult Viable	Adult Viable
	OE^2^: *Flag.caz*^*WT*^	75	Adult Viable	Death at P
	OE^2^: *Flag.caz*^*P398L*^	75	Death at P	Death at P
	OE^2^: *Flag.hFUS*^*WT*^	75	Adult Viable	Death prior to L3
	OE^2^: *HA.hFUS*^*WT*^	128	Death at P	Death at P
	OE^1^: *hFUS*^*P525L*^*-RFP.HA*	129	Death prior to L3	N/A
	OE^1^: *hFUS*^*WT*^*-RFP.HA*	129	Death prior to L3	N/A
	OE^1^: *hFUS*^*R524S*^*-RFP.HA*	129	Death at P	Death at P
	OE^2^: *Flag.hFUS*^*P525L*^	75	Death at P	Death at L3
**Hs:** ***C9orf72***	OE^2^: *G*_*4*_*C*_*2*_*-3*	123	Adult Viable	Adult Viable
	OE^2^: *G*_*4*_*C*_*2*_*-36*	123	Adult Viable	Adult Viable
	OE^2^: *GR-36*	123	Adult Viable	Adult Viable
	OE^2^: *GR-100*	123	Death prior to L3	N/A
	OE^2^: *PR-36*	123	Adult Viable	Adult Viable
	OE^2^: *PR-100*	123	Adult Viable	Adult Viable
**Hs:** ***SOD1*; Dm:** ***Sod1***	LOF: *Sod1*^*n1*^/+*	80	Adult Viable	Adult Viable
	LOF^2^: *Sod1-RNAi [Pembroke]*	122	Adult Viable	Adult Viable
	LOF^1^: *Sod1-RNAi [Hertford]*	122	Adult Viable	Adult Viable
	OE^1^: *hSOD1*^*WT*^	130	Adult Viable	Adult Viable
	OE^2^: *hSOD1*^*WT*^*. HA*	131	Adult Viable	Adult Viable
	OE^1^: *hSOD1*^*G85R*^	130	Adult Viable	Adult Viable
	OE^1^: *hSOD1*^*A4V*^	130	Adult Viable	Adult Viable
	OE^1^: *Sod1*	130	Adult Viable	Adult Viable

^1^Transgenesis: random insertion; ^2^Transgenesis: Φ-C31 site-specific insertion; ^*^Heterozygote; Hs, *Homo sapiens* (human); Dm, *Drosophila melanogaster* (fruit fly); N/A = Not Applicable; L3, third instar larval stage; P, pupal stage, LOF, loss of function; OE, overexpression.

Recently, considerable attention has been given to the toxic effect of cytoplasmic TDP-43 protein aggregates^71^. To this end, we next queried whether gain rather than loss of TDP-43 function is a modifying factor. Expression of wild-type human TDP-43 (hTDP-43^WT^) in muscle leads to adult flies that have climbing defects and are entirely flightless when compared to controls (Fig. 1). Flies also have a shortened life-span, therefore surviving less than a week post-eclosion. Notably, in combination with Gem3^BART^, hTDP-43 induced adult lethality with flies dying at the pupal stage. Furthermore, transgenes with higher expression levels and/or epitope-tagged versions of hTDP-43 (*hTDP-43*^*WT*^*.GFP*^*#10*^, weak expression; *hTDP-43*^*WT*^*.GFP*^*#16*^, strong expression; ref. ^72^) were found to induce death at the earlier third instar stage (L3) when combined with *Gem3*^*BART*^ in contrast to death at pupal stage when they were expressed alone (Table 1). This trend, which was also observed with an independently-generated line (*Flag.hTDP-43*^*WT*^, ref. ^73^; Table 1), shows that phenotypic enhancement is dependent on dose or modifications that interfere with protein structure. Muscle-directed expression of hTDP-43^CTF^, which mimics the major TDP-43 C-terminal fragment found in cytosolic aggregates of ALS patients, was found to induce lethality at the first instar larval stage most likely because the transgene is highly expressing^72^. This excluded its use for interaction analysis (Table 1). Importantly, overexpression of endogenous TBPH replicated the modifier effect of its human counterpart. Hence, whereas alone it induces death at the pupal stage, when combined with Gem3^BART^ it enhanced survival defects with flies dying earlier than the L3 stage (Table 1). Overall, these findings are suggestive of a genetic interaction between *Gemin3* and *TBPH* or its human homologue, *TDP-43*.

### Knockdown or overexpression of *caz/FUS* enhances *Gem3* mutant phenotypes

**Figure 2:**
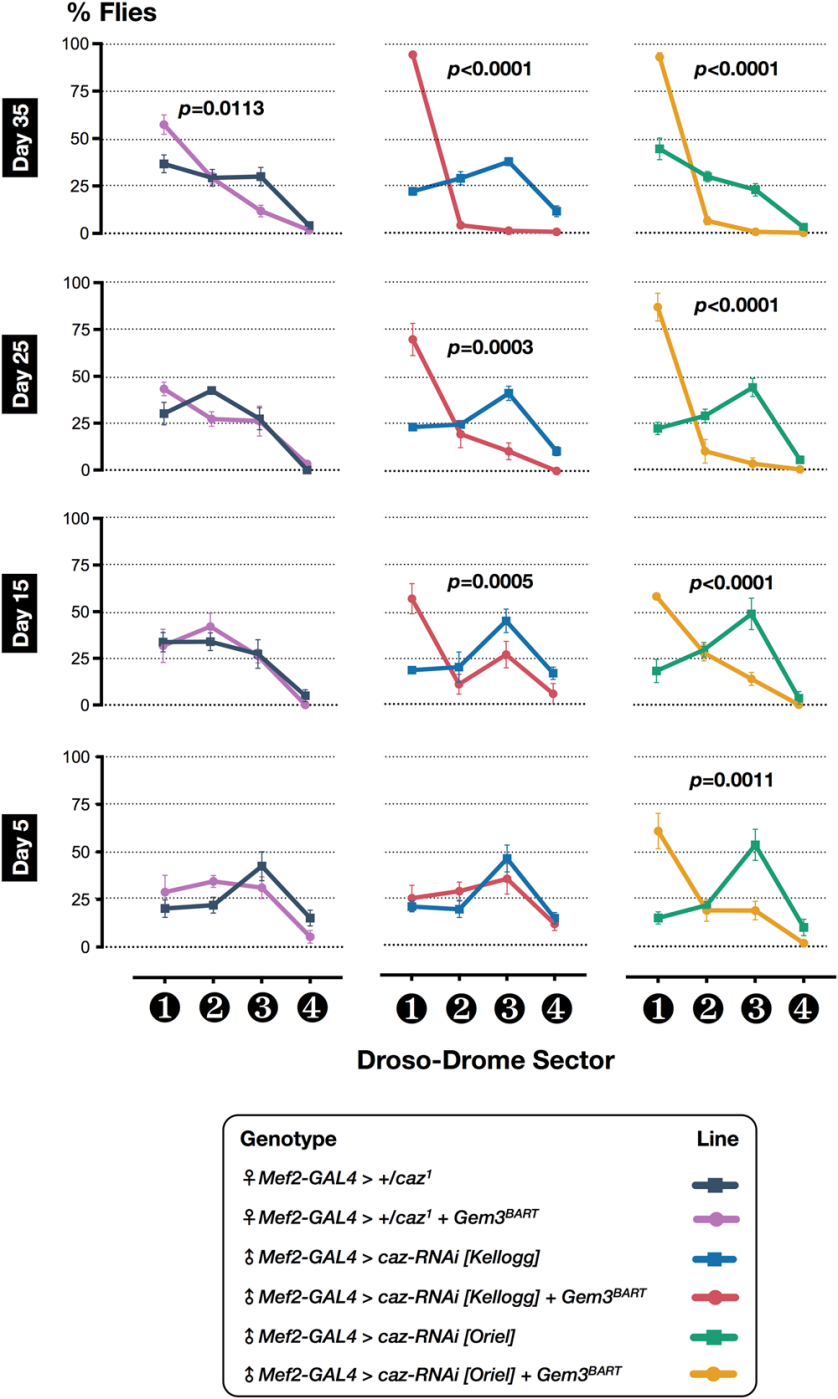
Loss of *caz* function impacts neuromuscular ability in *Gem3* mutant flies. *Left panel*: In a heterozygous *caz* deficient background, brought about by the *caz*^1^ null mutant, *Gem3*^*BART*^ flies develop flight defects at a late stage during adulthood. Hence, motor deficits become obvious only on day 35 post-eclosion. *Middle panel*: A greater reduction in Caz levels induced by a moderately-expressing RNAi transgene (*caz-RNAi [Kellogg]*) induced flight defects at an earlier stage (day 15 post-eclosion) and an increase in severity was observed with age. *Right panel*: A more pronounced age-dependent progressive decline in flight capacity can be brought about by knockdown mediated by a stronger RNAi transgene (*caz-RNAi [Oriel]*). Data presented are the mean ± S.E.M. of at least 4 independent experiments, and, for each time point measured, *n* ≥ 60 per genotype. Symbols indicate the sex of the genotype assessed: ♂ = males, and ♀ = females. Significance as tested by two-way ANOVA, followed by Bonferroni’s *post hoc* tests is indicated by the exact *p*-value.

FUS is another major RBP that is mutated in both familial (5%) and sporadic (<1%) ALS cases^10^. FUS is a highly-conserved protein possessing an N-terminal domain rich in glutamine, glycine, serine and tyrosine residues (QGSY region), a glycine-rich region, an RRM, multiple arginine/glycine/glycine (RGG) repeats in an arginine- and glycine-rich region, and a zinc finger motif at the C-terminus. Mutations cluster in the glycine-rich region and in the extreme C-terminus where the nuclear localisation signal is likely to reside^11^. Similar to TDP-43, mutant FUS is mislocalised to the cytoplasm where it forms ubiquitinated aggregates^74^. Considering that FUS and TDP-43 function in a common pathway with FUS acting downstream of TDP-43^75,76^, we hypothesised that Gemin3 is likely to have a functional relationship not only with TBPH/TDP-43 but also with FUS. To this end, we first tested whether haploinsufficiency of *cabeza* (*caz*), the *Drosophila* homologue of FUS, can induce motor deficits when placed in a *Gem3*^*BART*^ genetic background. Interestingly, we find a subtle yet statistically significant difference in motoric abilities at late adulthood (day 35 post-eclosion) in *caz* mutant heterozygous flies (*caz*^1^/+) that had muscle-restricted *Gem3*^*BART*^ expression compared to those that did not (Fig. 2). Subsequently, we asked whether phenotypic enhancement is dose-dependent. Hence, we induced muscle-specific RNAi-mediated knockdown of *caz* in wild-type versus Gem3^BART^ flies. We observed that a moderately-expressing RNAi transgene targeting the C-terminus (*caz-RNAi [Kellogg]*, Supplementary Fig. S1) induced flight defects as early as day 15 post-eclosion with flies then exhibiting an age-dependent progressive worsening in phenotype (Fig. 2). A stronger RNAi transgene targeting the same region but based on short hairpin microRNA (shRNA) technology (*caz-RNAi [Oriel]*, Supplementary Fig. S1) was capable of inducing motor defects at an earlier stage during adulthood (Fig. 2), further confirming that modification is dependent on Caz protein levels with a severe reduction inducing the highest impact.

**Figure 3:**
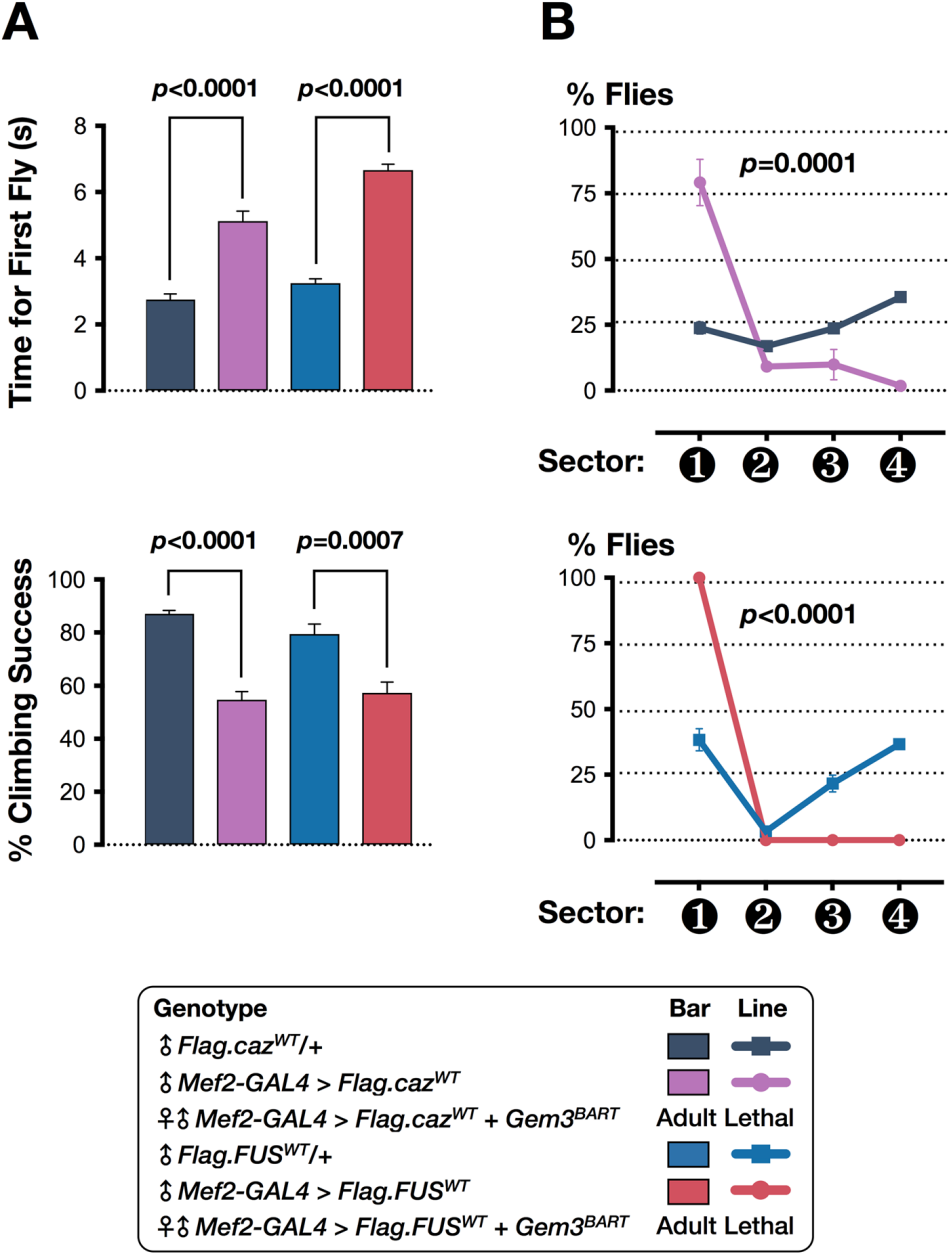
Gain of *caz*/*FUS* function in muscle impairs motor performance. (**A**) Overexpression of either *caz* or human *FUS* (*hFUS*) in muscle leads to adult flies with reduced mobility. Hence, on assessment, the first fly took significantly longer to reach a pre-set threshold. Furthermore, at a population level, climbing success was profoundly reduced. (**B**) Flies were in their majority non-fliers. Thus, when tested, a significant percentage dropped to the base (sector 1) of the Droso-Drome. Importantly, in combination with *Gem3*^*BART*^, muscle-specific overexpression of either *caz* or *hFUS* induced lethality prior to eclosion. Data presented are the mean ± S.E.M. of at least 4 independent experiments, and for each time point measured, *n* ≥ 60 per genotype. Symbols indicate the sex of the genotype assessed: ♂ = males, ♀ = females, and ♀♂ = males + females. Adult-viable flies were assessed at day 5 post-eclosion. Significance as tested by the unpaired *t*-test (**A**) and two-way ANOVA, followed by Bonferroni’s *post hoc* tests (**B**) is indicated by the exact *p*-value.

Similar to TDP-43, in addition to loss of nuclear function, a toxic gain of function due to the formation of cytoplasmic aggregates has been implicated as a predominant mechanism underpinning FUS-associated pathophysiology^74,77^. In this context, we questioned whether upregulation of *caz* or overexpression of human FUS (hFUS^WT^) can also act as enhancers when placed in a *Gem3* mutant background. In this regard, in a wild-type background, muscle-restricted increase in Caz protein levels was sufficient to induce both climbing and flight defects as early as day 5 post-eclosion (Fig. 3). However, in Gem3^BART^ flies, *caz*^*WT*^ upregulation induced lethality before eclosion with the majority of flies dying during the pupal stage (Table 1). Similarly, ectopic expression of hFUS^WT^ in muscles was enough to cause motor deficits in young adult flies when applied to a wild-type background (Fig. 3). In combination with Gem3^BART^, hFUS^WT^ overexpression remarkably induced death during early development (Table 1). A trend towards reduced survival was also observed in Gem3^BART^ flies upon expression of hFUS with a pathogenic mutation in the C-terminus (*hFUS*^*P525L*^), though not when expressing *caz*^*P398L*^, its equivalent in *Drosophila* (Table 1). Collectively, these findings show that either loss or gain of Caz/FUS function aggravate the motor and viability phenotypes of *Gem3* mutant flies, which is highly suggestive of a genetic association between *Gemin3* and *caz/FUS*.

### Expression of *C9orf72* repeat expansions has no effect on Gem3 mutant flies

We next sought to broaden our investigation by determining whether Gem3 mutant phenotypes are also induced by disruption of other major ALS-linked genes. *C9orf72* is the most frequently mutated gene in ALS. Enormous expansions of an intronic hexanucleotide repeat (GGGGCC, G_4_C_2_) cause a large portion of familial (25%) and sporadic (10%) ALS^10^. Transcripts containing repeats form intranuclear RNA foci that sequester nuclear proteins. In the cytoplasm, expanded RNA also undergoes repeat-associated non-AUG (RAN) translation to produce toxic dipeptide repeat (DPR) proteins^9^. Transgenic expression of a non-pathogenic repeat length (G_4_C_2_-3) in muscle tissue had no effect on neuromuscular function in either wild-type or *Gem3*^*BART*^ flies. Thus, at all time points assessed, flies were relatively healthy and no major differences were apparent between the two groups at any stage during their adult life (Fig. S2). Although muscle-restricted expression of 36 repeats (G_4_C_2_-36), previously shown to be neurotoxic^78^, caused flight defects in early adulthood, the phenotype in *Gem3*^*BART*^ flies was surprisingly identical to that of control flies with a wild-type background (Fig. S2). It is noteworthy that a similar outcome was observed in either background when assessing animals expressing two arginine-containing DPR proteins, glycine-arginine (GR-36) or proline-arginine (PR-100) (Fig. S2). In addition to confirming that repeats are damaging through the production of DPR proteins^78^, this result underscores that neither expanded repeats nor poly-GR/poly-PR proteins enhance *Gem3* loss-of-function.

### Loss rather than gain of Sod1 function enhances motor deficits in Gem3 mutant flies

**Figure 4:**
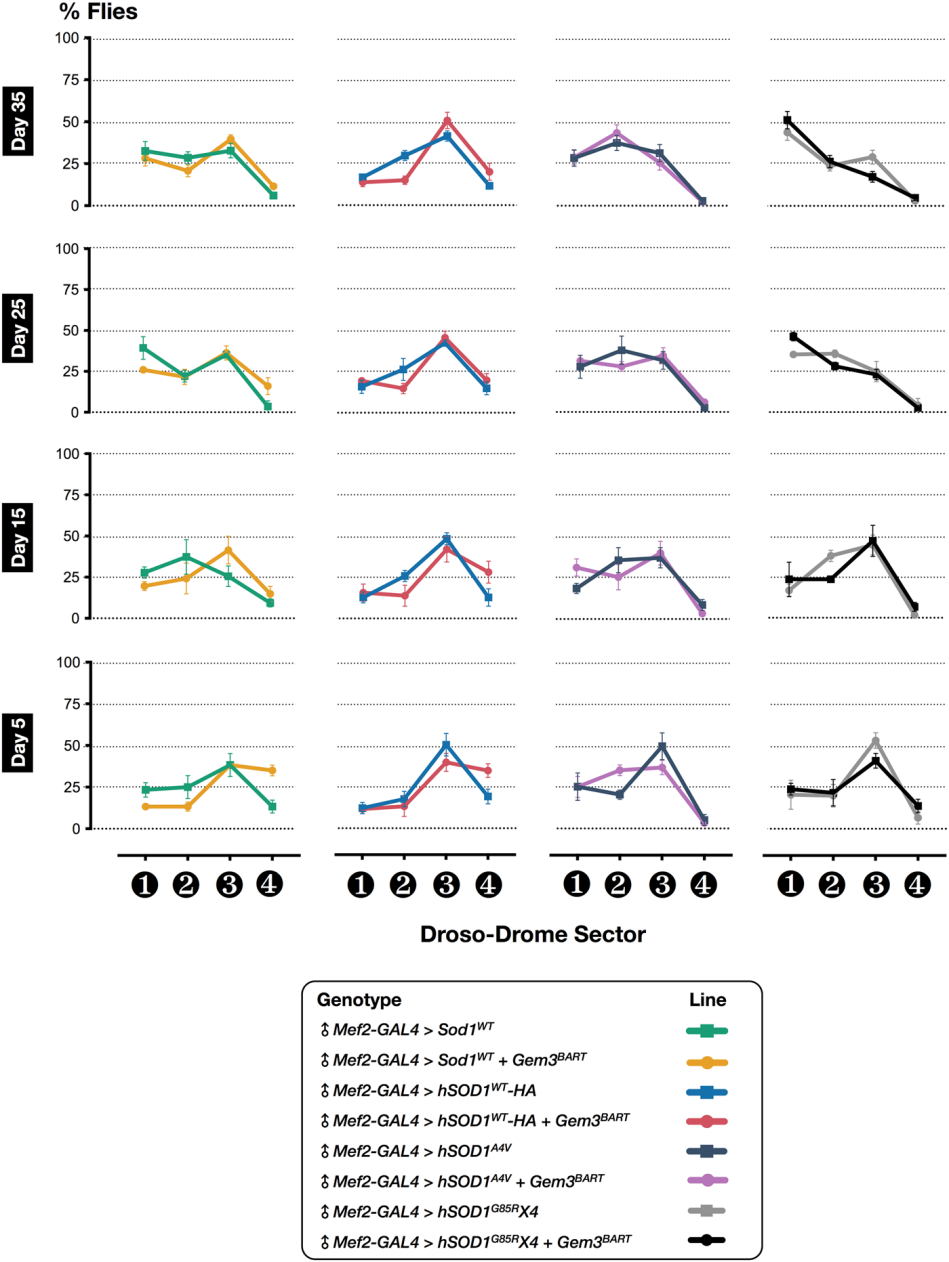
Sod1 gain-of-function has no effect on motor behaviour in Gem3 mutant flies. Overexpression of either *Sod1* or human *SOD1* (*hSOD1*) is not deleterious to animals with a marginal loss of Gem3 function (*left panel*). Overexpression of pathogenic hSOD1 A4V or G85R variants is equally not a damaging factor to Gem3 mutant flies (*right panel*). Organisms did not show differences in motor function at all time points assessed. Data presented are the mean ± S.E.M. of at least 4 independent experiments, and, for each time point measured, *n* ≥ 60 males (♂) per genotype.

**Figure 5:**
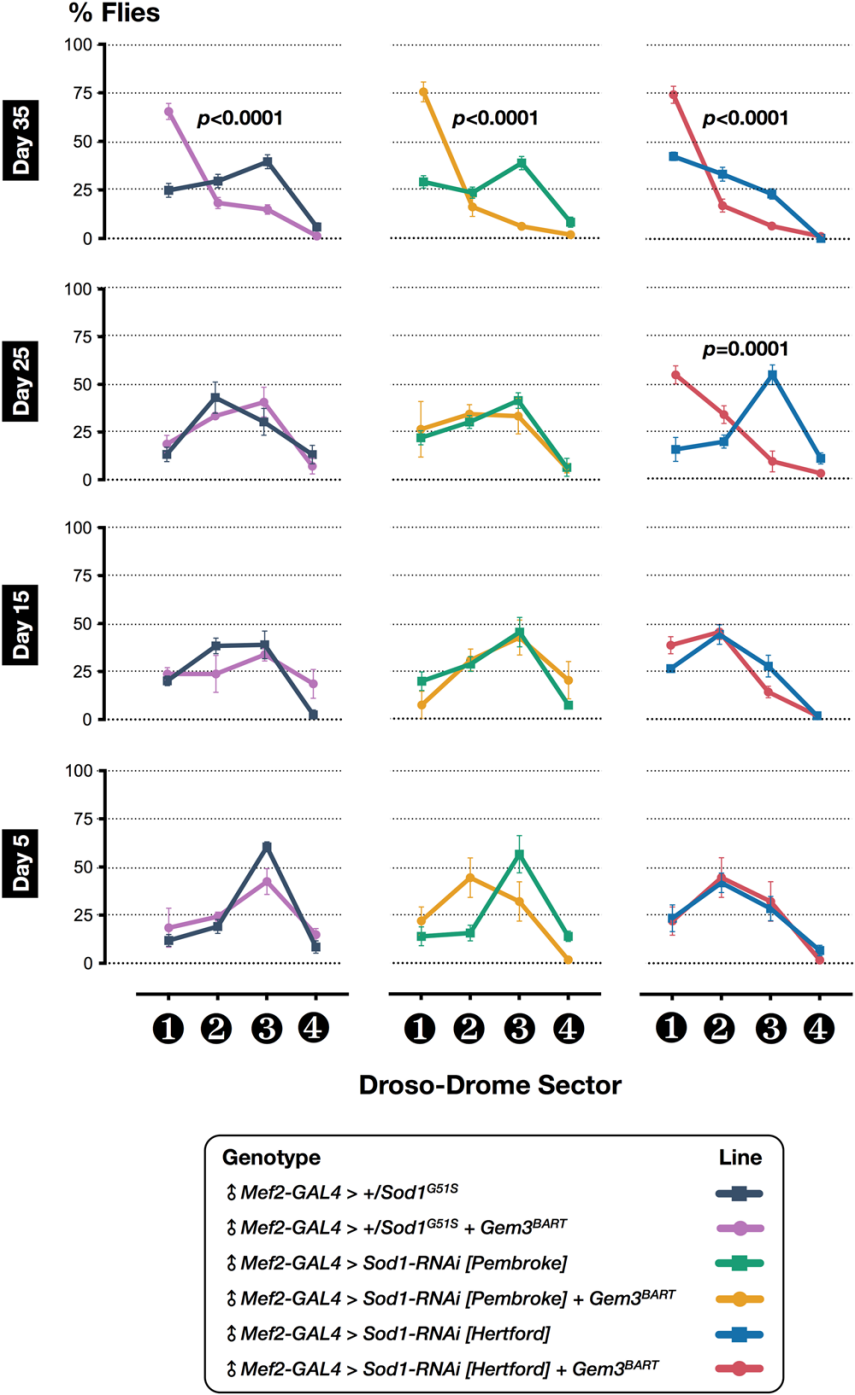
Loss of *Sod1* function impairs motor behaviour in *Gem3* mutant flies. Placed in a *Gem3*^*BART*^ genetic background, heterozygotes for an enzymatic null *Sod1* allele (+*/Sod1*^*G51S*^) only induce deficits in old age (*left panel*). A similar outcome is achieved in Gem3^BART^ flies with muscle-selective RNAi-mediated knockdown of *Sod1* (*middle panel*). The use of a stronger RNAi transgene causes defects at an earlier stage of adult life (*right panel*). Data presented are the mean ± S.E.M. of at least 4 independent experiments, and, for each time point measured, *n* ≥ 60 males (♂) per genotype. Significance as tested by two-way ANOVA, followed by Bonferroni’s *post hoc* tests, is indicated by the exact *p*-value.

The first ALS gene to be identified was *SOD1*, which encodes for the Cu-Zn superoxide dismutase, an abundant ubiquitously-expressed cytoplasmic enzyme. *SOD1* is the second most commonly mutated gene in ALS, contributing to 20% and 2% of familial and sporadic cases, respectively^10^. SOD1 performs an important antioxidant function by catalysing the conversion of highly reactive superoxide to hydrogen peroxide or oxygen. Nevertheless, neuromuscular degeneration is thought to be driven by one or more acquired toxicities of the mutant protein rather than loss of dismutase activity. Indeed, similar to TDP-43 and FUS, most ALS-causing SOD1 mutants form ubiquitinated cytoplasmic aggregates that are toxic to various cellular processes^79^. Against this backdrop, we investigated whether gain of Sod1 function is also capable of triggering motor dysfunction in *Gem3* mutant flies. We found that neither overexpression of *Drosophila* Sod1 nor ectopic expression of wild-type human SOD1 (hSOD1^WT^) had any negative effect in either a wild-type or a *Gem3* mutant background (Fig. 4). Expression of pathogenic variants including hSOD1^A4V^ or hSOD1^G85R^ gave a similar result, hence they were not damaging in either genetic background (Fig. 4). Interestingly, we were surprised to note that less than 50% reduction in enzymatic activity, brought about by heterozygosity for the missense allele *Sod1*^*G51S*^ (+*/Sod1*^*G51S*^)^80^, induced a prominent decrease in neuromuscular function during late adulthood in *Gem3* mutant flies, suggesting that these organisms are susceptible to oxidative stress when they get old. We confirmed this result through the use of muscle-specific RNAi-mediated loss of *Sod1* function in wild-type versus *Gem3*^*BART*^ flies. Hence, we show that an RNAi transgene targeting the C-terminus (*Sod1-RNAi [Pembroke]*, Supplementary Fig. S1) similarly provoked flight defects in adult flies aged to day 35 post-eclosion (Fig. 5). Motor defects became apparent at an even earlier stage (day 25 post-eclosion) when we made use of a stronger RNAi transgene targeting the same region but having a longer hairpin sequence (*Sod1-RNAi [Hertford]*, Fig. 5 and Supplementary Fig. S1). Overall, these findings demonstrate that *Gem3* mutant phenotypes are hastened by *Sod1* loss-of-function rather than by gain-of-function, hence allowing us to uncover a genetic interaction between *Gemin3* and *Sod1*.

### TDP-43 or FUS gain of function enhance muscle atrophy and suppresses neuromuscular junction overgrowth in *Gem3* mutant flies

**Figure 6:**
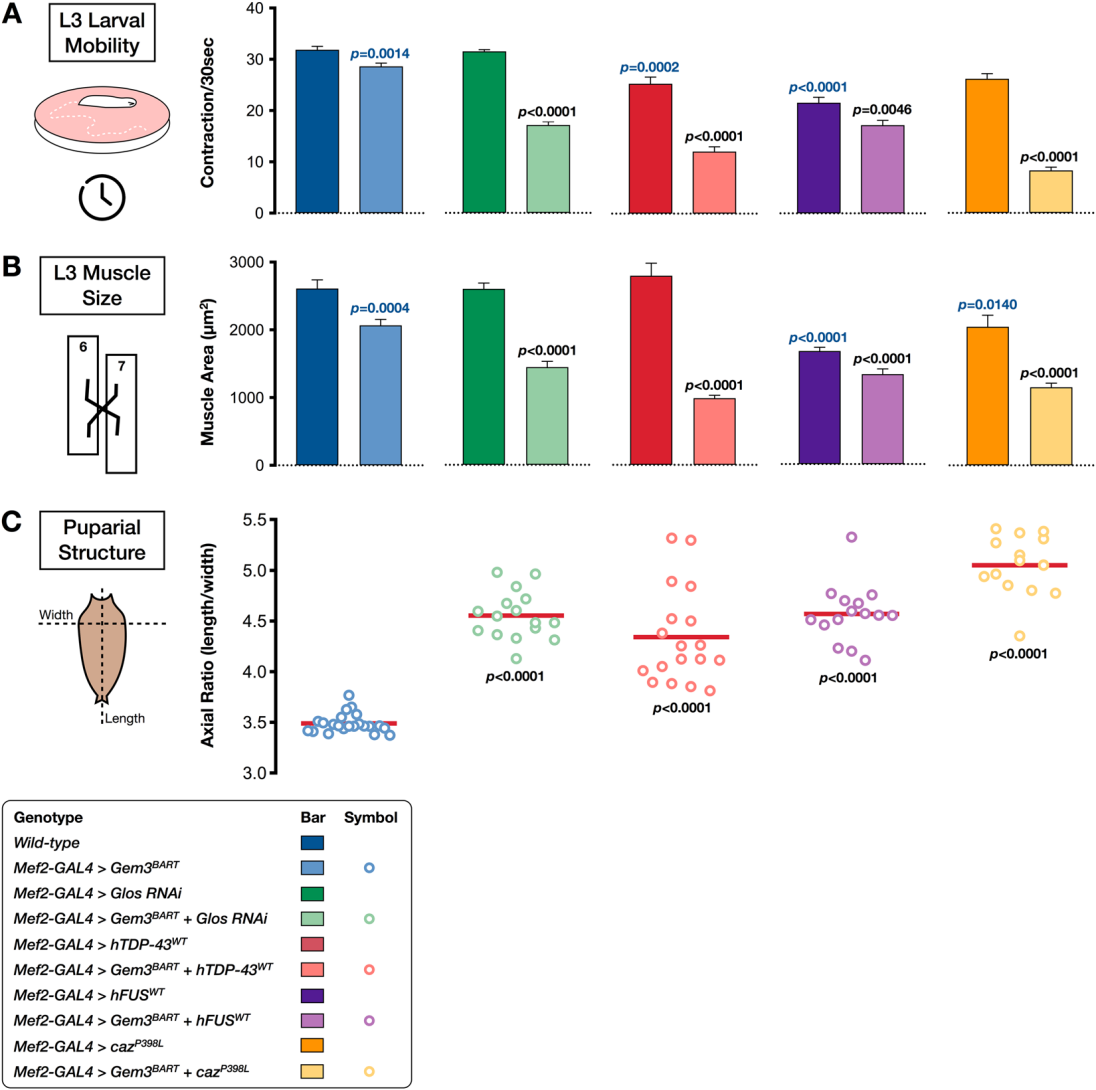
Overexpression of hTDP-43 or hFUS/*caz*^*P398L*^ in Gem3 mutant flies causes early mobility defects, reduced muscle size and aberrant puparial structures. (**A**) Third instar larvae with muscle-specific Gem3^BART^ expression have a significant reduction in velocity compared to wild-type controls. This deficit was profoundly enhanced on overexpression of *hTDP-*4*3*^*WT*^, *hFUS*^*WT*^ or *caz*^*P398L*^. A similar result was obtained on knockdown of *Gaulos* (*Glos RNAi*), which served as a positive control. In the absence of Gem3^BART^, only overexpression of *hTDP-43*^*WT*^ or *hFUS*^*WT*^ was found to induce a decrease in larval mobility compared to wild-type animals. (**B**) Compared to the wild-type control, *Gem3*^*BART*^ larvae also show a significant reduction in muscle size that dramatically worsens on *Glos* knockdown or upon overexpression of *hTDP-43*^*WT*^, *hFUS*^*WT*^, or *caz*^*P398L*^, all directed to muscle. In a wild-type background, muscle size was also found reduced in larvae with *Glos* knockdown or those overexpressing *FUS/caz*^*P398L*^. (**C**) Sluggish larval behaviour leads to the formation of puparia that have a significantly higher axial ratio (defined as length/width) compared to the baseline offered by *Gem3*^*BART*^ larvae. In (**A–C**) data presented are the mean ± S.E.M. of at least 3 independent experiments, and *n* ≥ 15 per genotype. Equal number of male and female larvae were used in each assay. Significance as tested by the unpaired *t*-test is indicated by the exact *p*-value, shown either in blue (comparison to wild-type larvae) or black (comparison to *Gem3*^*BART*^ larvae).

Above we showed that upregulation of *caz* or *TBPH*, in flies with *Gem3* loss of function, induced adult lethality. Overexpression of the respective human homologue gave an analogous outcome. We next investigated whether the neuromuscular function of these animals is perturbed prior to their death. Gem3^BART^ animals devoid of any genetic modifying factor(s) eclose normally and neuromuscular function is relatively unperturbed in adult flies. Surprisingly, when analysing larval crawling, we observed a slight yet significant decline in mobility in *Gem3*^*BART*^ larvae compared to their wild-type counterparts (Fig. 6A). Notably, this difference can be explained by a substantial difference in muscle surface area between *Gem3* mutant and control larvae. Thus, the former had a pronounced reduction in muscle size (Fig. 6B). We asked whether these phenotypes are amenable to modification by genetic factors. To this end, we introduced *Gaulos* RNAi in *Gem3* mutants. Gaulos (Glos) was recently identified as the *Drosophila* orthologue of Gemin4^67,81^, a putative co-factor of Gemin3^82^. Remarkably, muscle-driven Glos reduction caused a further decline in both the locomotor ability (Fig. 6A) and muscle size of *Gem3*^*BART*^ larvae (Fig. 6B), hence confirming that the phenotypes can be genetically enhanced. Importantly, we demonstrate that compared to the baseline provided by *Gem3*^*BART*^ larvae, ectopic expression of either hTDP-43 or hFUS was responsible for an additional degree of sluggishness in larvae as demonstrated by their less frequent movements (Fig. 6A). Interestingly, the difference was reflected in muscle size, hence muscle atrophy was greatly enhanced upon TDP-43 or FUS gain-of-function (Fig. 6B). Upregulation of *TBPH* causes *Gem3*^*BART*^ flies to die before the third instar larval stage (Table 1), hence precluding assessment of flies. Upregulation of *caz* in *Gem3*^*BART*^ flies induces larval mobility defects but has no effect on muscle size (data not shown). However, overexpression of the pathogenic variant *caz*^*P398L*^ in *Gem3*^*BART*^ flies was found to mirror the neuromuscular phenotypes observed on TDP-43/FUS gain of function in the same genetic background (Fig. 6A,B).

**Figure 7:**
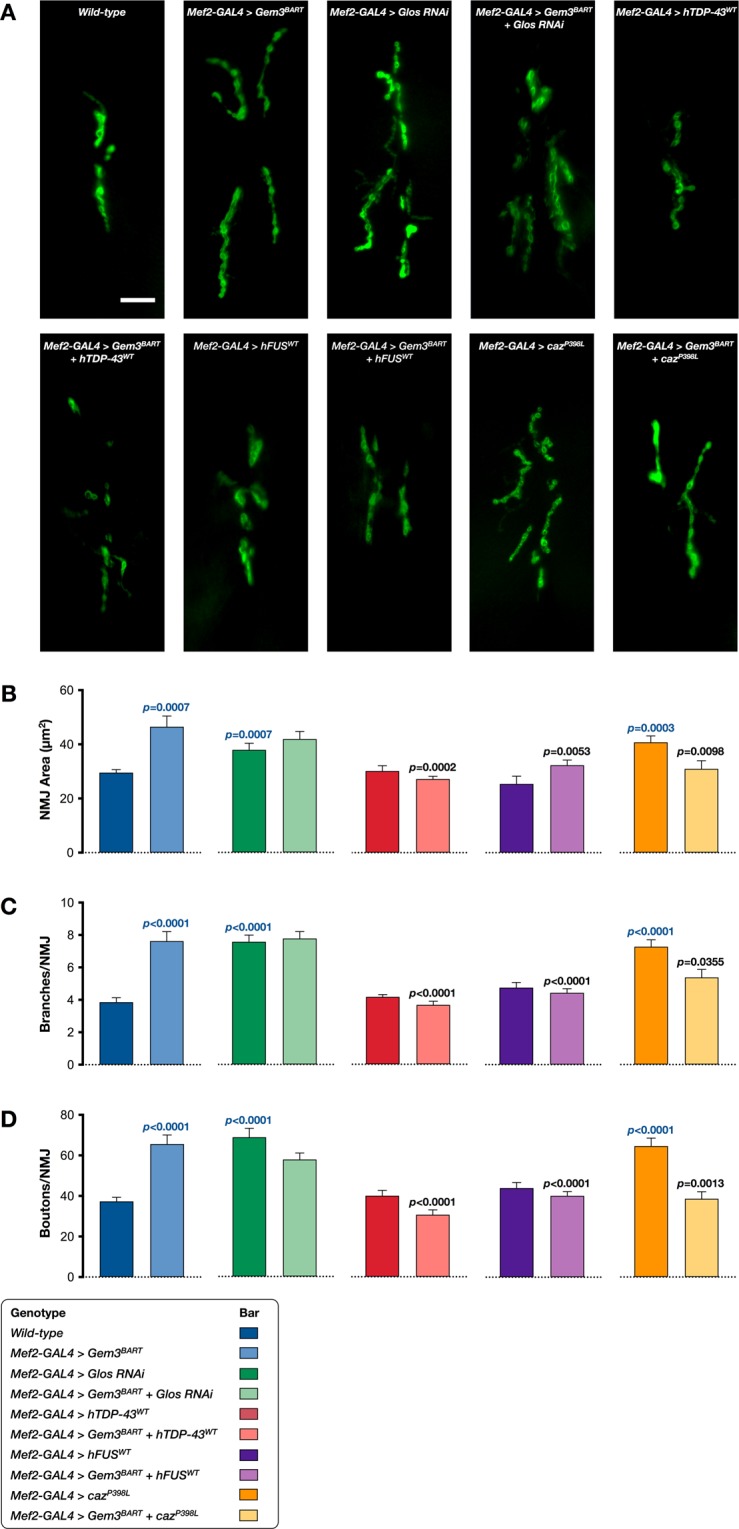
Overexpression of *hTDP-43* or *hFUS*/*caz*^*P398L*^ suppresses NMJ expansion in Gem3 mutant flies. (**A**) Representative images of NMJs innervating ventral longitudinal muscles 6 and 7 in third instar larvae stained with post-synaptic anti-DLG antibody (scale bar = 10 μm). Visual inspection reveals that compared to wild-type, muscle-directed expression of Gem3^BART^ induces an overgrown NMJ morphology that is restored on overexpression of *hTDP-43*^*WT*^, *hFUS*^*WT*^ and, to a lesser degree, *caz*^*P398L*^. An overgrowth phenotype is also obvious on *Glos* knockdown (applied singularly or combined with Gem3^BART^) or in flies expressing *caz*^*P398L*^. These observations were confirmed upon quantification of NMJ area (**B**), number of branches per NMJ (**C**) and number of boutons within a single NMJ (**D**). In (**B–D**) data presented are the mean ± S.E.M. and *n* ≥ 18 per genotype. Equal number of male and female larvae were assessed. Significance as tested by the unpaired *t*-test is indicated by the exact *p*-value, shown either in blue (comparison to wild-type larvae) or black (comparison to *Gem3*^*BART*^ larvae).

Consequent to muscle atrophy and the subsequent decline in muscle power, all genotypes assessed failed to contract adequately during pupariation. Hence, in a *Gem3*^*BART*^ genetic background, *Glos* knockdown (serving as a positive control) or overexpression of *hTDP-43*/*hFUS*/*caz*^*P398L*^, all induced a puparial axial ratio that was significantly higher than the baseline observed in *Gem3* mutant flies devoid of any genetic manipulation (Fig. 6C). Finally, we assessed the morphology of the neuromuscular junction (NMJ). Muscle-directed expression of Gem3^BART^ causes an appreciative expansion of the NMJ (Fig. 7A), hence, parameters including area (Fig. 7B), number of branches (Fig. 7C) and bouton numbers (Fig. 7D) were all significantly elevated compared to the wild-type control. This phenotype was similar to that previously reported for flies with homozygous *Gem3* loss-of-function in all tissues^65^. NMJ overgrowth was also observed on *Glos* knockdown and upon expression of *caz*^*P398L*^, both of them directed to muscle (Fig. 7). No deviations from the wild-type NMJ parameters were however seen on expression of either hTDP-43 or hFUS in muscle (Fig. 7). Interestingly, in a Gem3^BART^ background, expression of *hTDP-43*, *hFUS* and, to a slightly lower degree, *caz*^*P398L*^ (but not *Glos* RNAi) suppresses the NMJ overgrowth phenotype associated with Gem3 loss-of-function, with key NMJ morphology parameters reduced to the wild-type range (Fig. 7). In sum, these observations strengthen the evidence favouring the possibility that *Gemin3* acts together with *TBPH/TDP-43* and *caz/FUS* within a genetic pathway that influences viability and neuromuscular function.

### Self-association of full-length Gemin3 explains loss of function induced by the Gem3^BART^ mutant

**Figure 8:**
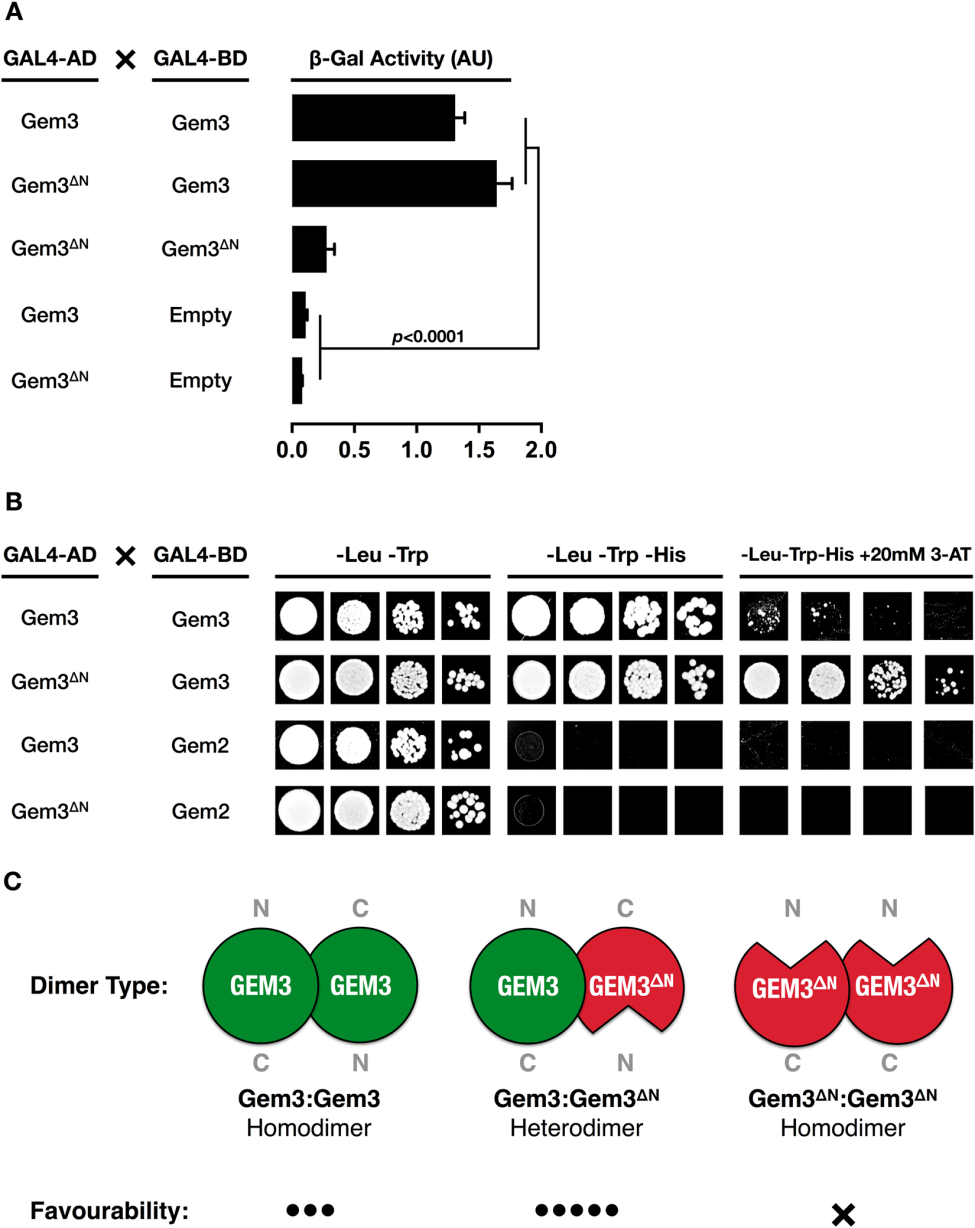
Full-length Gemin3 interacts not only with itself but also with *Gem3*^*ΔN*^. (**A**) Measurement of β-galactosidase activity was used to assay the expression of the GAL1-LacZ reporter gene that is produced by the combinations of the indicated proteins. Full-length Gemin3 interacts with itself or with its N-terminal truncated form (Gem3^ΔN^). Gem3^ΔN^ is not capable of self-binding. An empty vector served as a negative control. Individual bars represent the mean ± S.E.M. β-galactosidase activity of 3 independent experiments. Significance as tested by one-way ANOVA is indicated by the exact *p*-value. (**B**) The reporter strain carrying yeast two-hybrid plasmids expressing the indicated proteins was spotted in serial dilutions on –Leu–Trp–His plates in the presence of 20 mM 3-AT. Under these conditions, Gem3-Gem3 interaction is unfavourable whereas the Gem3-Gem3^ΔN^ association remains favourable. Interaction between Gem3 or Gem3^ΔN^ with Gemin2 served as a negative control. At least 3 independent experiments were performed, and the result of one representative is shown. (**C**) Model for Gemin3 dimerisation. *Left*, Gemin3 monomers are hypothesised to bind to each other in the reverse direction. *Middle*, The Gem3^ΔN^ mutant is a truncated version of Gemin3 lacking the N-terminus, which hosts the helicase domains. In the presence of Gem3^ΔN^, wild-type:mutant dimers are favoured more than wild-type:wild-type dimers. *Right*, A model predicting self-association in the opposite orientation infers that Gem3^ΔN^ is not self-binding, hence, mutant:mutant dimers do not form. Abbreviations: N, N-terminus; C, C-terminus.

In conclusion, we wished to gain insights into the mode of action of the *Gem3*^*BART*^ allele, a hypomorphic or weaker version of the *Gem3*^*ΔN*^ mutant. The latter differs from wild-type Gemin3 in that it lacks the N-terminus which hosts the helicase domains^68^. Previously, we have shown that expression of *Gem3*^*ΔN*^ mimics Gem3 knockdown and, together, the genetic alterations cause lethality. This allowed us to conclude that expression of *Gem3*^*ΔN*^ in a wild-type background induces a loss of Gemin3 function^64^. We hypothesised that *Gem3*^*ΔN*^ interacts with its wild-type full-length counterpart and in so doing, it interferes with its function. This model assumes that Gemin3 is capable of self-association for which the evidence is presently lacking. Through a yeast two-hybrid assay, we confirm this model, hence, we show that *Drosophila* Gemin3 is able to strongly interact with itself (Fig. 8A). Although this property is lost with *Gem3*^*ΔN*^, remarkably, we demonstrate that *Gem3*^*ΔN*^ is capable of binding to its full-length counterpart (Fig. 8A).

We next questioned whether the *Gem3*^*ΔN*^-Gem3 interaction is stronger than the one between wild-type Gemin3 monomers, hence favouring the capture of endogenous Gemin3 into non-functional oligomers. To this end, we re-performed the yeast two-hybrid assay in the presence of 3-amino-1,2,4-triazole (3-AT). 3-AT competitively inhibits imidazole glycerol-phosphate dehydratase, a histidine (His) biosynthetic enzyme, thus limiting His synthesis^83,84^. We demonstrate that yeast containing both the Gem3 bait and the *Gem3*^*ΔN*^ prey were capable of growing on –Leu–Trp–His selective plates at 20 mM 3-AT (Fig. 8B), thus indicating that the two-hybrid Gem3-Gem3^ΔN^ interaction is strong enough to overcome the growth inhibitory effect of 3-AT in the medium. In summation, these findings are first supportive of Gemin3 self-binding. Second, they allow us to postulate that the dominant-negative nature of *Gem3*^*ΔN*^ arises from its most-favourable binding to endogenous Gemin3. This is predicted to titre wild-type Gemin3 into non-functional dimers or oligomers.

## Discussion

In this study, we sought to determine whether a functional interaction exists between Gemin3, a core SMN complex component, and major ALS-associated proteins. Focusing on motor behaviour, muscle mass, NMJ structure and survival, which are all profoundly affected in motor neuron disease, we show that disruption of either TBPH/TDP-43 or Caz/FUS enhance muscle defects but are able to suppress NMJ morphology deficits, both induced by Gemin3 loss-of-function. We also found that depletion of Sod1 has an enhancing effect on neuromuscular function in old age. In addition to highlighting shared pathways most likely involving aspects of ribostasis and oxidative stress, our findings reinforce the link between SMA and ALS. Importantly, they extend our knowledge on the function of Gemin3, showing for the first time that it self-interacts, which is a property that makes it prone to loss of function.

Defective chaperoning of spliceosome assembly and/or missplicing have long been known to have a major role in the pathophysiology of motor neuron degeneration. A plethora of *in vivo* studies unequivocally show that snRNP assembly defects and the consequential missplicing events can induce the selective motor phenotype that is typical in SMA patients (reviewed in ref. ^6^). In this regard, we have recently shown that, in *Drosophila*, perturbation of snRNP biogenesis factors pICln or Tgs1 causes motor deficits that mirror those brought about by loss of SMN or select Gemins including Gemin3^61^. This corroborates earlier findings demonstrating that knockdown of pICln or U1 snRNP leads to MND-like phenotypes in zebrafish^85,86^. Notably, by discovering an interaction between Gemin3 and either pICln or Tgs1^61^, we underscored that these factors participate in a common pathway that most likely centres on the synthesis of snRNPs which form the backbone of the spliceosome. Here, we widen our findings by uncovering a genetic association between Gemin3 and two RBPs with important roles in diverse aspects of RNA metabolism, namely TDP-43 and FUS. Our data suggest that Gemin3, TDP-43 and FUS function in overlapping pathways that influence viability, muscle mass, NMJ morphology and motoric ability.

Considering the roles of Gemin3, TDP-43 and FUS at different points in the life of the spliceosome, we speculate that the intersecting pathways are vital for the correct splicing of mRNAs, which we believe is a main contributor to the health and optimal function of the neuromuscular system. In support, although its exact activities in snRNP assembly remain unclear, Gemin3 is indispensable for this process *in vivo*^60^. Considering TDP-43 and FUS, long before their implication in ALS, these two proteins were reported to influence pre-mRNA splicing or interact with known splicing factors^87,88^. Both RBPs were later shown to bind to predominantly UG-rich sequences in RNA transcripts, regulating the expression and alternative splicing of multiple yet distinct target genes, particularly those with exceptionally long introns^89–93^. Thus, loss of TDP-43 or FUS in cell lines or mouse brain leads to splicing defects that are mostly different for either factor^89,90,92,94,95^. Even splicing of snRNP components is altered based on studies in sporadic ALS patient-derived lower motor neurons that had nuclear TDP-43 depletion^96^ or FUS knockdown in a human cell line^97^. Overexpression of ALS-causing TDP-43 or FUS mutants, which cause neuromuscular phenotypes in mice, were also found to induce aberrant RNA splicing^98,99^. Similarly, *Drosophila* with knockout or overexpression of TBPH exhibit splicing alterations^100^. Interestingly, expression levels of several snRNAs were found significantly increased in brains of flies overexpressing human TDP-43^101^ and decreased in fibroblasts derived from ALS patients with FUS mutations or FUS transgenic mice^19^. Notably, in an ALS mouse model, an endogenous C-terminal domain mutation in TDP-43 was recently reported to induce a gain of splicing function. Hence, splicing activity of TDP-43 was modified in such a way that it leads to the excision of otherwise normally conserved exons, thereby, termed ‘skiptic exons’^102^. Pathogenic TDP-43 or FUS mutations are also known to affect splicing in a gain-of-toxic-function manner by mislocalising snRNPs, SMN and splicing factors (PSF and NeuN) to the cytosol^18,19,86,103,104^.

It is interesting to note that whereas gain of TDP-43 or FUS function were both shown to enhance Gemin3 motor deficits, we observed that loss-of-function was consequential only for FUS. This can be explained by the more intimate relationship of FUS with the SMN complex. In this respect, in addition to U1 (and U11) snRNP components^19,103^, SMN complex members including SMN and Gemin3 were shown to be integral members of the FUS interactome^19^. It is plausible that the SMN complex might collaborate with FUS in an as yet unknown snRNP-related function, which is disrupted by deviations from normal Caz/FUS levels. Thus, ALS-causative mutations in FUS were found to strengthen the interaction with SMN potentially sequestering SMN and, most probably, its associates, from their normal localisation and function^19^. It is important to note that we do not exclude the possibility that Gemin3 cooperates with TDP-43 and FUS in other steps of RNA metabolism including transcription or RNA transport given that all three factors are known to participate in either process^11,12,28,60,105^.

Gem3 mutant phenotypes were not hastened by *SOD1* or *C9orf72* gain-of-function. This allows us to infer that the genetic interaction between *Gemin3* and *TBPH/TDP-43* or *caz/FUS* is specific. Nonetheless, reduced levels of *Sod1* brought about by heterozygosity for a *Sod1* mutant or RNAi-mediated knockdown were surprisingly found to induce motor deficits in *Gem3* mutant flies during late adulthood. Oxidative stress is exacerbated by age^106^ and paucity of Sod1^107–109^. Notably, snRNP assembly function of the SMN complex was found to be inhibited by oxidative stress in a dose-dependent manner^110^. This observation adds to the plethora of evidence showing that oxidative stress perturbs RNA metabolism (reviewed in ref. ^111^). Thus, it is reasonable to speculate that oxidative stress is a modifying factor for Gemin3 function in snRNP synthesis as part of the SMN complex.

Ectopic overexpression of the helicase core deletion mutant Gem3^ΔN^ in a wild-type background induces phenotypes that overlap those resulting from Gemin3 loss-of-function. In this regard, either overexpression of Gem3^ΔN^ or RNAi-mediated knockdown of Gemin3, both targeted to muscle tissue, was shown previously to disrupt motor behaviour. Applied simultaneously, these two genetic manipulations were found to cause lethality^64^. Thus, the evidence favours the possibility that Gem3^ΔN^ interferes with endogenous Gemin3 to induce loss of function, hence acting as a dominant-negative mutant or a Muller’s antimorph. Here, we show that Gemin3 is capable of self-interaction and Gem3^ΔN^ retains the ability to interact with its wild-type counterpart. Importantly, analysis of interaction strength demonstrates that wild-type:mutant dimers are favoured more than wild-type:wild-type dimers (Fig. 8C). Based on these findings, we predict that Gem3^ΔN^ titres endogenous Gemin3 into non-functional dimers or oligomers. Sequestration of Gemin3 can perturb SMN complex stoichiometry in addition to inhibiting the participation of Gemin3 in SMN complex-related activities including snRNP assembly or recycling.

The formation of wild-type:mutant dimers or even oligomers can potentially hinder the catalytic activity of Gemin3, thus raising the question of whether dimerization of Gemin3 is a prerequisite for its ATPase-dependent RNP chaperoning activities. Self-interaction that is independent of the RNA substrate is rather unusual for members of the DEAD-box RNA helicase family. It has been reported in prokaryotes for *Escherichia coli* RhIB^112^, *Thermus thermophilus* Hera^113^, *Bacillus subtilis* CshA^114^ and cyanobacteria CrhR^115^. In this context, our findings add Gemin3 to the growing list of eukaryotic DEAD-box RNA helicases that also have a self-interaction property including transcriptional regulators DDX5/p68 and DDX17/p72^116^. Domain analysis of both *T. thermophilus* Hera and *B. Subtilis* CshA revealed that efficient dimerization is dependent on protein regions other than the those hosting the two highly-conserved RecA-like helicase domains which are required for RNA substrate binding and catalytic activity^113,114^. For *E. coli* RhIB, cyanobacteria CrhR^115^ and eukaryotic DDX5 or DDX17, a large part of the conserved core was required for self-association^112,116^, a finding that also applies for Gemin3. To this end, we show that, alone, the C-terminal domain of Gemin3 is incapable of self-binding, hence, the formation of Gem3^ΔN^ homodimers is an unfavourable reaction (Fig. 8C). However, interaction is observed in the presence of the N-terminus, making the formation of Gem3^ΔN^:Gem3 dimers a highly favourable reaction. This indicates that self-interaction requires that the full-length protein is present in at least one of the two monomers, thus raising the possibility that Gemin3 monomers bind to each other in the reverse direction. This model warrants future investigation through further molecular and structural studies.

Given the functional interaction of ALS-linked TBPH/TDP-43, Caz/FUS and Sod1 with Gemin3, which itself is intimately associated with the SMA-causative SMN, our work adds to the substantial collection of evidence supporting convergence of the molecular mechanisms of two major MNDs. Although we speculate that defects in RNA metabolism might be central to the pathophysiology of ALS and SMA, further investigation of the mechanistic overlaps is now possible in a genetically-tractable model organism. Importantly, given our findings, we propose Gemin3 as a candidate for modifying motor neuron degeneration.

## Materials and Methods

### Flies

Flies were cultured on food consisting of sugar, corn meal, yeast, and agar in plastic vials at an incubation temperature of 25 °C under 12 hours day/night cycles. The wild-type strain was *w*^*1118*^. For adult-based assays, male flies were used except where indicated. In instances where females were used, flies were virgins. For larval-based assays, equal number of male and female larvae were assessed. Inducible transgenes were expressed via the bipartite GAL4/upstream activation sequence (UAS) system (reviewed in ref. ^117^). Muscle-exclusive expression was achieved through the use of the *Mef2*-GAL4 driver^118^. The *Gem3*^*BART*^ allele (*UAS.Gem3*^*BART*^) was generated previously by transposition of the *Gem3*^*ΔN*^ transgenic allele into a repressive region on chromosome 2^68^. The *UAS.Glos-IR*^*DEX*^ (*Glos RNAi*) transgene was described and characterised previously^67^. *TBPH*^*Δ23*^ and *caz*^*1*^ are small deletions that partially remove the coding and 5’ sequence of *TBPH*^73^ and *caz*^75^, respectively. They are considered as null alleles of the respective gene. The *Sod1*^*G51S*^ mutant (also known as *Sod1*^*n108*^ or *Sod1*^*n1*^) carries an EMS-generated missense mutation in the *Sod1* gene that disrupts dimer contact^119^. *Sod1*^*G51S*^ homozygotes were reported to be null for superoxide dismutase activity. In *Sod1*^*G51S*^ heterozygotes, superoxide dismutase activity was reported to be close to 40%^80^. The RNAi transgenic constructs, *UAS.TBPH-RNAi [Trinity]* (ID: 38377), *UAS.TBPH-RNAi [Merton]* (ID: 38379), *UAS.TBPH-RNAi [Maudlin]* (ID: 104401), *UAS.caz-RNAi [Kellogg]* (ID: 100291), *UAS.caz-RNAi [Oriel]* (ID: 330388), *UAS.Sod1-RNAi [Hertford]* (ID: 31551), and *UAS.Sod1-RNAi [Pembroke]* (ID: 108307) were obtained from the Vienna *Drosophila* Resource Center, Austria^120^, and were described previously^73,121,122^. The provenance of the various UAS transgenes for *TBPH*, *hTDP-*4*3*, *caz*, *hFUS, Sod1* and *hSOD1* is referenced in Table 1. All *C9orf7*2-related transgenic lines were obtained from the Bloomington *Drosophila* Stock Center (NIH P40OD018537) at Indiana University, USA and were characterised previously^123^. Combination of the various genetic tools including GAL4 drivers, alleles, and transgenes was performed according to standard genetic crossing schemes.

### Mobility assays

Mobility assays in larvae and adult flies were conducted as described previously^67^. In brief, third instar larvae were first placed on a 0.7% agar plate. Subsequently, the number of forward body wall contractions exhibited by the organism in 30 seconds were counted. Each larva was assessed three times before an average was taken. To assess climbing performance in adult flies, two empty polystyrene tubes were vertically joined by tape facing each other. Flies (15–20) were then transferred into the lower tube and allowed to acclimatize. Flies were then gently tapped down to the bottom of the vial. The time for the first fly within a group to cross an 8 cm threshold was first measured. Consequently, the number of flies per group, that can climb above the 8 cm mark by 10 seconds, was determined. For each group of flies, four trials were performed. A minimum of four groups were assayed per genotype.

### Flight assay

Flight performance was assessed as detailed previously^61,67^. This assay made use of the Droso-Drome apparatus, which consists of a 1 L glass bottle coated with an alcohol-based sticky fluid, and divided into 4 sectors, of 5 cm each, spanning a total height of 20 cm. In short, flies first underwent a ‘warm-up’ by inducing negative geotaxis in an empty tube for 6 times. Organisms were then dropped into the Droso-Drome to induce flight. The number of flies stuck to each sector was next counted, divided by the total number of flies dropped and multiplied by 100 to generate the percentage number of flies per sector. Fight ability correlates with the height or sector in which flies are distributed, hence, fly percentages that are skewed towards the lower sectors of the Droso-Drome are indicative of reduced flight capacity.

### Puparial axial ratios

Length and width of puparia were first measured from still images. As reported previously^61,67^, calculation of puparial axial ratios involved dividing the length by the width of the puparia.

### Immunohistochemistry

The same immunohistochemistry procedures described previously^65^ were followed. Briefly, body wall muscles of wandering third instar larvae were dissected in phosphate buffered saline (PBS), fixed in 4% paraformaldehyde in PBS and washed in PBS + 0.1% Triton X-100 (PBT). Tissues were then stained overnight at room temperature by mouse anti-Discs large antibody (1:1000; Developmental Studies Hybridoma Bank, University of Iowa, USA). On the following day, tissues were washed in PBT and stained overnight at room temperature with anti-mouse Alexa Fluor 488-conjugated secondary goat antibody (1:50) and Alexa Fluor 546-conjugated Phalloidin (1:50). After a final wash in PBT, the samples were mounted in 90% glycerol with anti-fade. Imaging was performed with Optika B-600TiFL microscope (20x or 40x objectives).

### Analysis of muscle size and NMJ morphology

ImageJ software (NIH) was used to quantify both muscle and NMJ area. The former comprised of both ventral longitudinal muscles 6 and 7 derived from abdominal segments 2–4 whereas the latter constituted the postsynaptic region on the same muscles stained by the anti-Discs large antibody. Branch number was determined by counting the number of arborisations containing at least two boutons within a single NMJ. To determine, bouton numbers, all boutons were counted within a single NMJ.

### Yeast two-hybrid assays

Two-hybrid assays were performed as described previously^67^. Briefly, baits and preys were obtained by PCR amplification of cDNA and ligation into the *pAS∆∆* and *pACT2st* vectors, respectively^124^. Primer sequences and PCR regimes are available upon request. The cDNA clones for *Gemin2* (LD47479) and *Gemin3* (LD05563) were obtained from the *Drosophila* Genomics Resource Centre (Indiana University, USA). Gem3^ΔN^ was synthesised as described previously^65^. The bait *pAS∆∆* construct containing the protein sequence fused in frame with the GAL4 DNA binding domain (GAL4-BD) was used to transform the CG1945 strain, which was then selected on –Trp plates. The prey *pACT2st* construct containing the protein sequence fused in frame with the GAL4 activation domain (GAL4-AD) was used to transform the Y187 strain, which was then selected on –Leu plates. Mating of bait and prey strains was achieved overnight on rich yeast extract peptone dextrose (YPD) plates and –Trp –Leu plates were used to select diploids containing bait/prey combinations. Protein-protein interactions were screened by spotting serial dilutions on –Trp–Leu–His plates. Where indicated, 30 mM 3-amino-1,2,4-triazole (3-AT) was added to the medium to assess interaction strength. Incubations were performed at 28° C for 3 to 5 days. The β-galactosidase assay was used to quantify yeast two-hybrid interactions. Cells were grown in –Trp–Leu selective medium to an OD_600_ = 0.5–1.0. Activity was measured from extracts as reported previously^125^.

### Statistical analysis

Values are presented as means ± S.E.M. unless otherwise indicated. The unpaired *t*-test was used to compare measures between 2 groups whereas one-way ANOVA was applied for multiple comparisons with the control. Two-way ANOVA, followed by Bonferroni’s *post hoc* test, was used to determine differences between 2 groups in the percentage number of fliers (sectors 2–4) vs. non-fliers (sector 1) in the flight assay (GraphPad Prism v8.0.1). Differences were deemed statistically significant if *p* < 0.05, and when this is the case, the exact *p*-value is presented.

## Supplementary information

Supplementary Information

## Data Availability

All data generated or analysed during this study are included in this published article.
