# Supplementary material for: SMN complex member Gemin3 self-interacts and has a functional relationship with ALS-linked proteins TDP-43, FUS and Sod1
Source: Sci Rep. 2019 Dec 10;9:18666. doi: 10.1038/s41598-019-53508-4 (PMC6904755; doi:10.1038/s41598-019-53508-4)
Supplement: Supplementary file 1 — Supplementary Information [file 41598_2019_53508_MOESM1_ESM.pdf]

SMN complex member Gemin3 self-interacts and has a functional relationship with ALS-linked proteins TDP-43, FUS and Sod1

Rebecca Cacciottolo[1,2,3], Joanna Ciantar[2,3], Maia Lanfranco[1,2,3], Rebecca M. Borg[1,2,3], Neville Vassallo[2,3], Rémy Bordonné[1] & Ruben J. Cauchi[2,3,*]

[1]*Institut de Génétique Moléculaire de Montpellier, CNRS-UMR 5535, Université de Montpellier, Montpellier, France*

[2]*Department of Physiology and Biochemistry, Faculty of Medicine and Surgery, University of Malta, Msida, Malta*

[3]*Centre for Molecular Medicine and Biobanking, Biomedical Sciences Building, University of Malta, Msida, Malta*

* Corresponding Author

E-mail: ruben.cauchi@um.edu.mt

SUPPLEMENTARY FIGURE S1

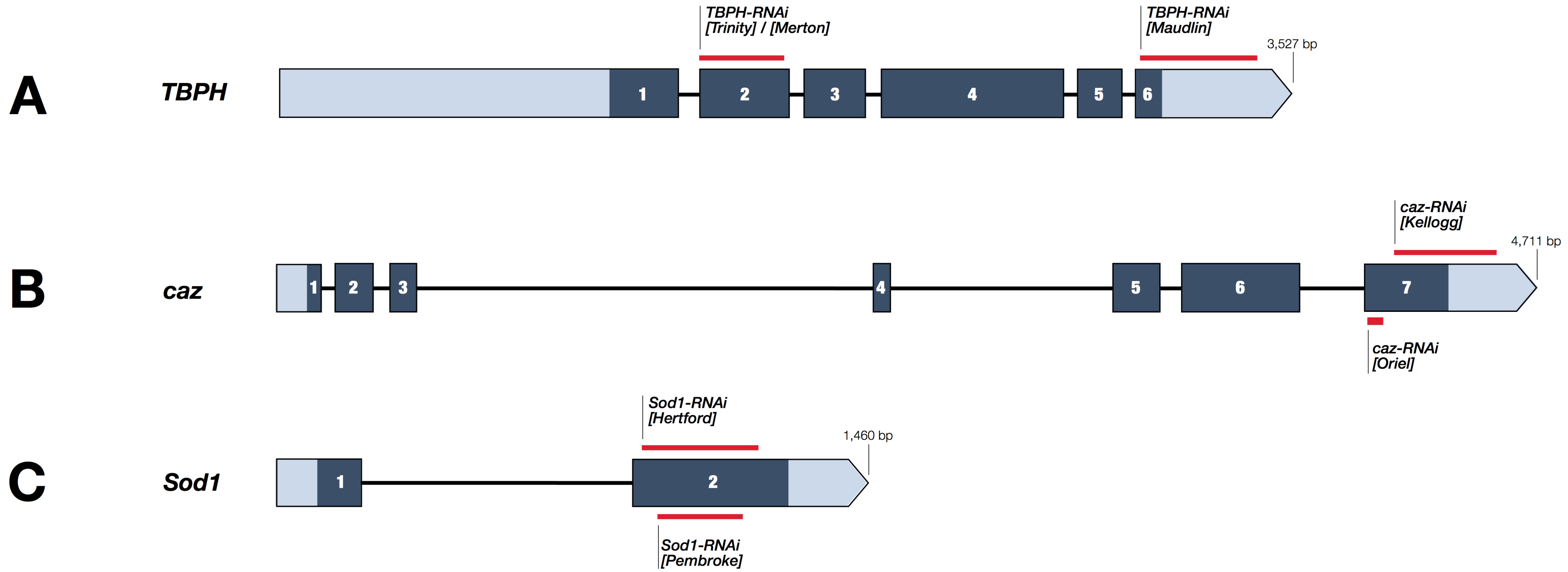

Figure S1. RNAi constructs utilised in the study. (**A**) The six-exon *TBPH* gene produces a 3.5 Kbp transcript (*TBPH-RC*, ID: FBtr0089625). *TBPH-RNAi [Trinity]* (chromosome 3 random insert) and TBPH-RNAi *[Merton]* (chromosome 2 random insert) are inducible RNAi constructs that have a 301 bp fragment derived from exon2 (annotated in red) as an inverted repeat (IR). A 392 bp fragment spanning exon6 and the 3' untranslated region or UTR (annotated in red) defines the IR of *TBPH-RNAi [Maudlin]* (chromosome 2 site-specific insert). (**B**) The seven-exon *caz* gene produces a 4.7 Kbp mRNA transcript (*caz-RB*, ID: FBtr0074217) that was targeted by two inducible RNAi transgenes. The *caz-RNAi [Kellogg]* transgene (chromosome 2 site-specific insert) consists of an IR composed of a 294 bp fragment spanning exon7 and the 3' UTR (annotated in red). The *caz-RNAi [Oriel]* transgene (chromosome 2 site-specific insert) consists of a short hairpin making use of a 21 bp targeting sequence derived from exon7 (annotated in red) that is embedded into a micro-RNA (miR-1) backbone. This strategy was shown to induce effective gene knockdown in both germline and somatic tissues[1]. (**C**) The two-exon *Sod1* gene produces a 1.46 Kbp mRNA transcript (*Sod1-RD*, ID: FBtr0333559). The *Sod1-RNAi [Hertford]* transgene (chromosome 3 random insert) consists of an IR composed of a 345 bp fragment derived from exon2 (annotated in red). The *Sod1-RNAi [Pembroke]* transgene (chromosome site-specific insert) consists of an IR composed of a 255 bp fragment also derived from exon2 (annotated in red). In (**A-C**) the transgenic constructs are attached to 10 copies of UAS sites to enhance RNAi efficiency[2].

SUPPLEMENTARY FIGURE S2

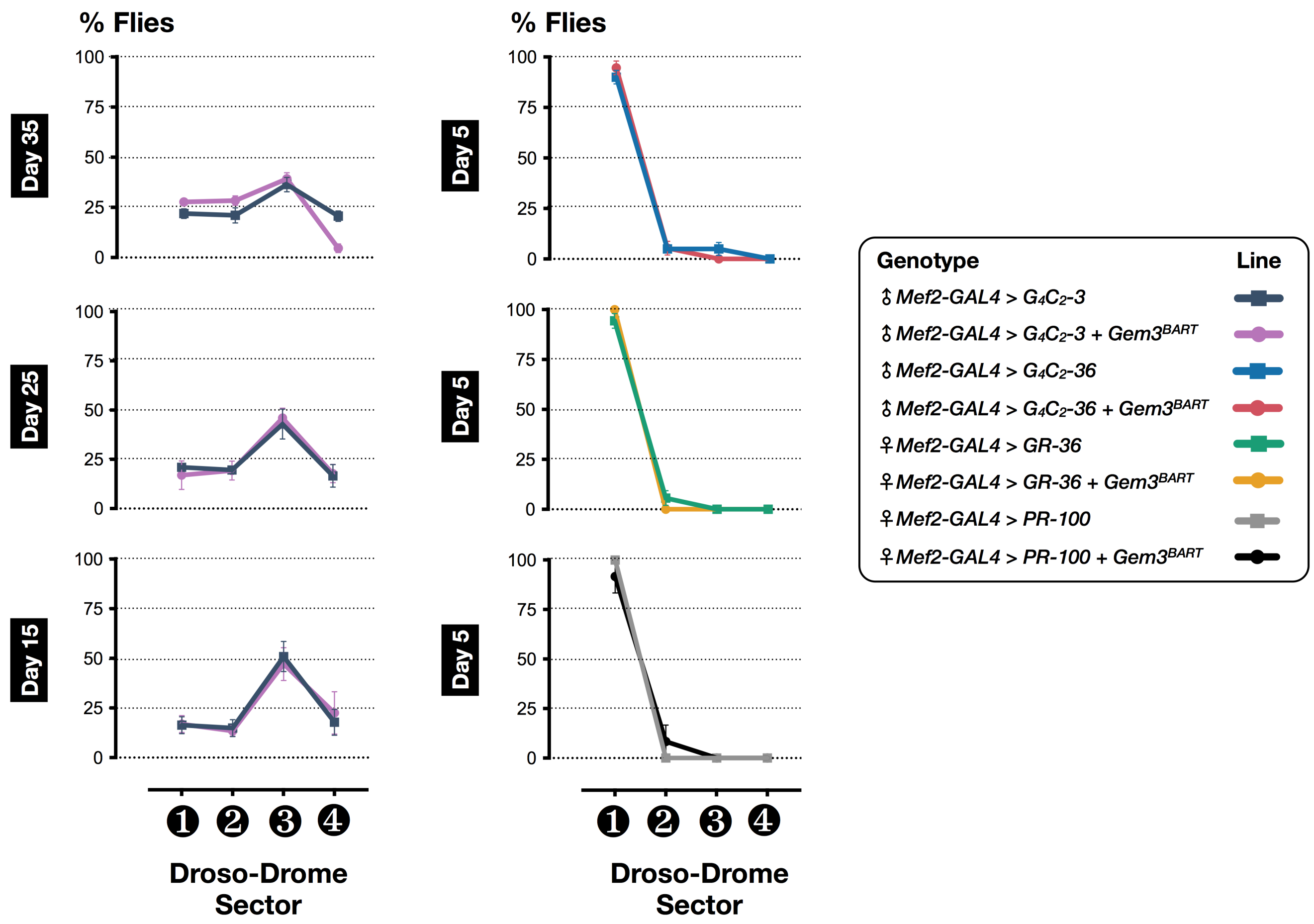

Figure S2. ***C9orf72*** **expanded hexanucleotide repeats and DPR proteins are not modifying factors in a Gem3 mutant.** No difference was observed in wildtype vs. *Gem3*^{BART} animals expressing normal length (G_4C_2-3) or expanded (G_4C_2-36) hexanucleotide repeats, the latter reducing motor function in young adults. Expression of glycine-arginine (GR-36) or proline-arginine (PR-100) DPR proteins was also found to induce motor defects at an early stage but, again, no disparities were apparent between the wild-type and *Gem3* mutant flies. Data presented are the mean ± S.E.M. of at least 4 independent experiments, and, for each time point measured, $n \geq 60$ per genotype. Symbols indicate the sex of the genotype assessed: ♂ = males, and ♀ = females.

SUPPLEMENTARY FIGURE S3

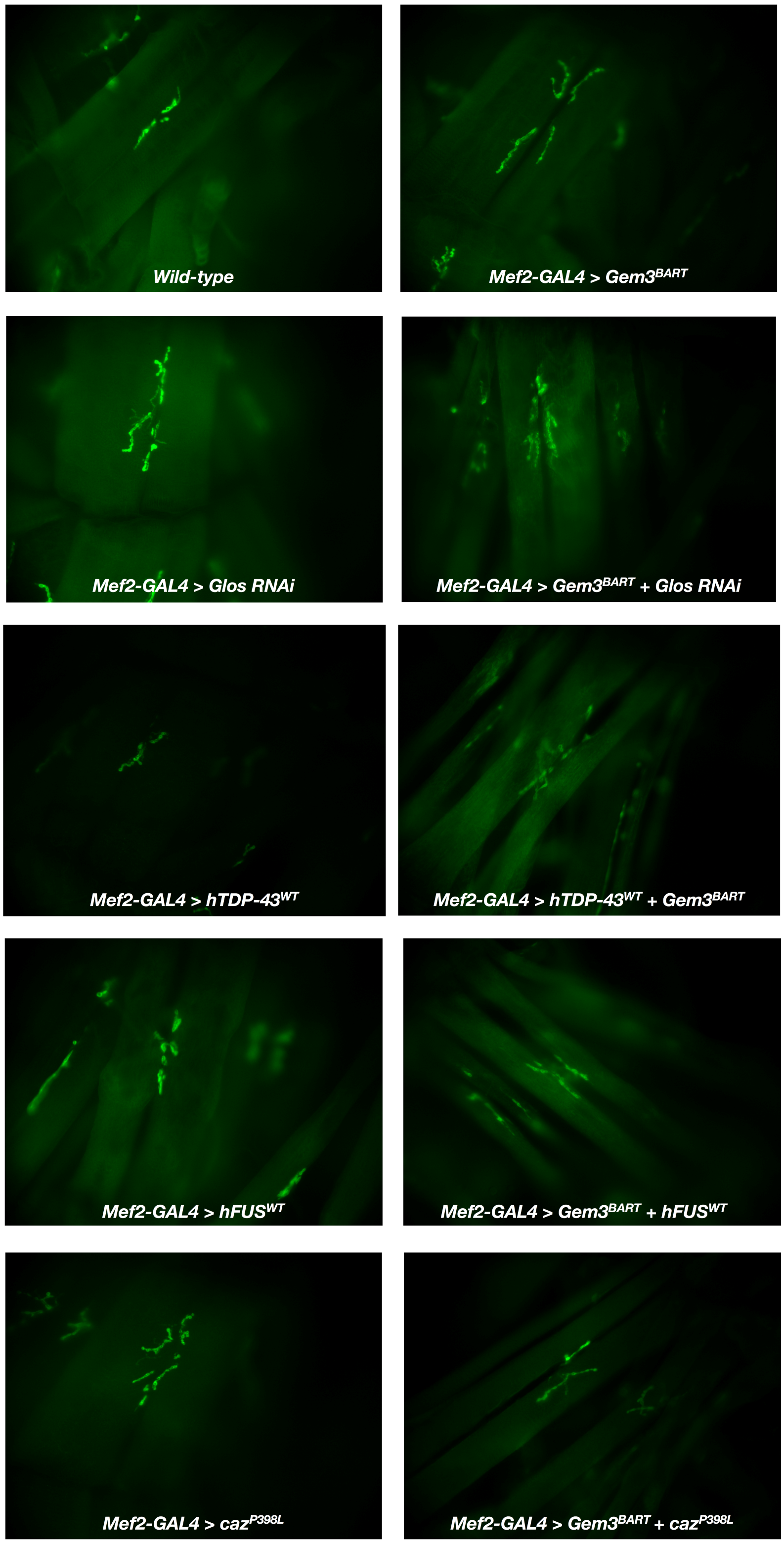

Figure S3. Uncropped and unedited (no adjustments in fluorescent signal intensity) images of neuromuscular junctions displayed in Fig. 7A.

SUPPLEMENTARY FIGURE S4

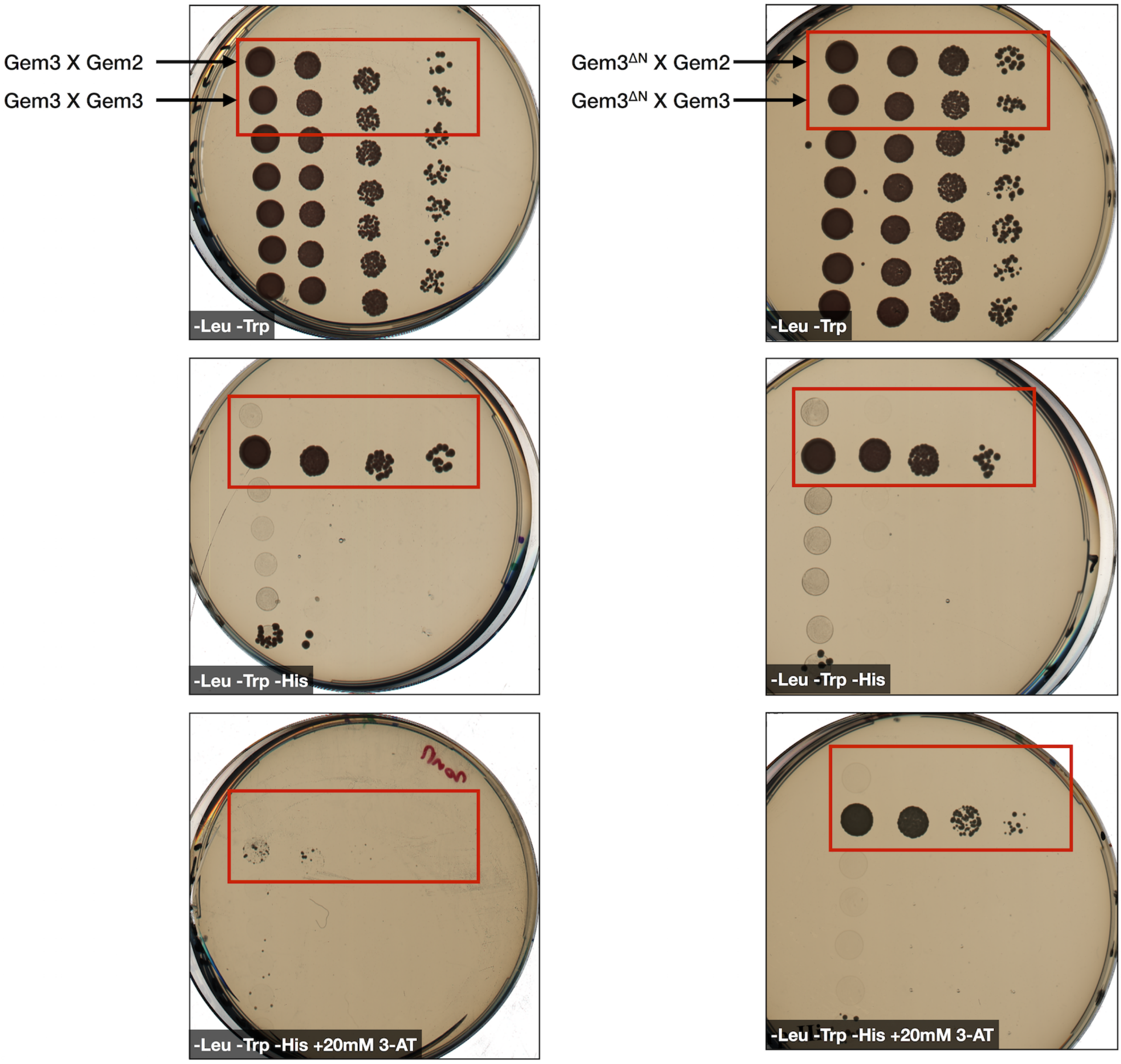

Figure S4. Uncropped and unedited images of yeast two-hybrid assays displayed in Fig. 8B.

REFERENCES

1 Ni, J. Q. *et al.* A genome-scale shRNA resource for transgenic RNAi in Drosophila. *Nat Methods* **8**, 405-407, doi:10.1038/nmeth.1592 (2011).

2 Dietzl, G. *et al.* A genome-wide transgenic RNAi library for conditional gene inactivation in Drosophila. *Nature* **448**, 151-156 (2007).
